# NAFLD: Mechanisms, Treatments, and Biomarkers

**DOI:** 10.3390/biom12060824

**Published:** 2022-06-13

**Authors:** Fatiha Nassir

**Affiliations:** Division of Gastroenterology and Hepatology, Department of Medicine, University of Missouri, Columbia, MO 65212, USA; nassirf@health.missouri.edu

**Keywords:** non-alcoholic fatty liver disease (NAFLD), metabolic-associated fatty liver disease (MAFLD), non-alcoholic steatohepatitis (NASH), metabolic-associated steatohepatitis (MASH), liver, mitochondria, sirtuins, lipotoxicity, reactive oxygen species (ROS), biomarkers

## Abstract

Nonalcoholic fatty liver disease (NAFLD), recently renamed metabolic-associated fatty liver disease (MAFLD), is one of the most common causes of liver diseases worldwide. NAFLD is growing in parallel with the obesity epidemic. No pharmacological treatment is available to treat NAFLD, specifically. The reason might be that NAFLD is a multi-factorial disease with an incomplete understanding of the mechanisms involved, an absence of accurate and inexpensive imaging tools, and lack of adequate non-invasive biomarkers. NAFLD consists of the accumulation of excess lipids in the liver, causing lipotoxicity that might progress to metabolic-associated steatohepatitis (NASH), liver fibrosis, and hepatocellular carcinoma. The mechanisms for the pathogenesis of NAFLD, current interventions in the management of the disease, and the role of sirtuins as potential targets for treatment are discussed here. In addition, the current diagnostic tools, and the role of non-coding RNAs as emerging diagnostic biomarkers are summarized. The availability of non-invasive biomarkers, and accurate and inexpensive non-invasive diagnosis tools are crucial in the detection of the early signs in the progression of NAFLD. This will expedite clinical trials and the validation of the emerging therapeutic treatments.

## 1. Introduction

Nonalcoholic fatty liver disease (NAFLD) is one of the most common causes of liver diseases, and its prevalence continues to increase worldwide [1]. The estimated annual medical costs directly attributed to NAFLD exceed €35 billion in four European countries (United Kingdom, France, Germany, and Italy) and $100 billion in the United States [2]. NAFLD is a spectrum of liver diseases that occur in the absence of other known causes, such as excess alcohol use. Since NAFLD is a metabolic disease, it has been recently renamed metabolic-associated fatty liver disease (MAFLD) [3]. The name MAFLD recognizes the disease as an independent disease entity and eliminates the absence of excess alcohol use criteria from the definition. To avoid nomenclature confusion, I will use the nomenclature NAFLD in this review. NAFLD includes hepatic steatosis with more than 5% of liver weight consisting of fat. NAFL may progress to nonalcoholic steatohepatitis (NASH), a more severe form of NAFLD with steatosis, inflammation, and cellular damage. NAFLD is among the leading etiologies for hepatocellular carcinoma (HCC) and liver transplantation [4]. The disease is linked to various extrahepatic disorders such as cardiovascular complications [5]. NAFLD affects ~24% of the general population [6] and has become an epidemic in parallel with the obesity epidemic. NAFLD is linked to obesity and type 2 diabetes (T2D) [6]. The disease affects up to 70% of overweight and more than 90% of morbidly obese people [7]. The strongest predictor of mortality in patients with MASH is hepatic fibrosis [8]. NAFL and NASH can also occur in lean subjects [9]. Asians, compared with non-Asian individuals, tend to have more lobular inflammation and higher grades of ballooning compared with other ethnicities [10]. In the Asian population, fat accumulation can occur at lower body mass [9]. In addition, ethnic disparity in the prevalence of NAFLD has been observed. NAFL and NASH are higher in Hispanics, intermediate in Whites, and lowest in Black people, but the underlying causes are unclear [10,11,12]. NAFLD prevalence is also increasing in children and young adolescents [13]. No pharmacological therapy is available to treat NAFLD, specifically. The reason might be that NAFLD is a multifactorial disease with a limited understanding of the pathogenic mechanisms involved and the absence of accurate non-invasive biomarkers. This review discusses the mechanisms involved in the pathogenesis of NAFLD, the current diagnostic tools, sirtuins SIRTs as potential therapeutic targets, and the non-coding RNAs as emerging biomarkers for the disease.

## 2. Pathogenesis of NAFLD

### 2.1. Liver Cells and NAFLD

The liver has a unique role in lipid metabolism as it represents a site for lipid uptake, synthesis, oxidation, and distribution of lipids to peripheral tissues. The cell population in the liver includes parenchymal cells (hepatocytes ~78% of the liver’s total cell population) [14]. Non-parenchymal cells include liver sinusoidal endothelial cells (LSECs), Kupffer cells (KCs), hepatic stellate cells (HSCs), and hepatic NK cells [14,15]. While hepatocytes have been associated with the liver’s primary function, such as lipid metabolism, KCs play a key role in liver inflammation [14,15]. KCs are resident macrophages that account for about 30% of sinusoidal cells [16] and 80% to 90% of macrophages in the human body [17,18]. Upon liver injury, KCs are activated to release inflammatory cytokines and chemokines contributing to the pathogenesis of NAFLD [18]. Liver inflammation is regulated by the balance between proinflammatory M1 KCs and anti-inflammatory M2 KCs [19]. The liver is exposed to various substances, such as nutrients and gut-derived bacterial products, via the portal circulation, which are eliminated by KCs [20]. KCs produce various inflammatory cytokines, including TNF-α, IL-1β, IL-6, IL-12, IL-18, and chemokines [18]. HSCs are at rest under normal circumstances; however, in response to inflammation and hepatocyte damage caused by lipotoxicity, they become activated and transform into myofibroblast-like cells, secreting increased collagen and causing fibrosis [21,22]. Little is known about the contribution of LSEC lipotoxcity to the progression of NAFLD. Lipotoxicity of LSEC may lead to decreases in both nitric oxide and an increase in reactive oxygen species (ROS) levels, resulting in oxidative stress and NASH [23]. The contribution of the different cells in the liver to NAFLD and the communication signals between parenchymal and non-parenchymal cells to the disease progression are still evolving [18,21,22,23].

### 2.2. Mechanisms for NAFLD Pathogenesis

**Figure 1:**
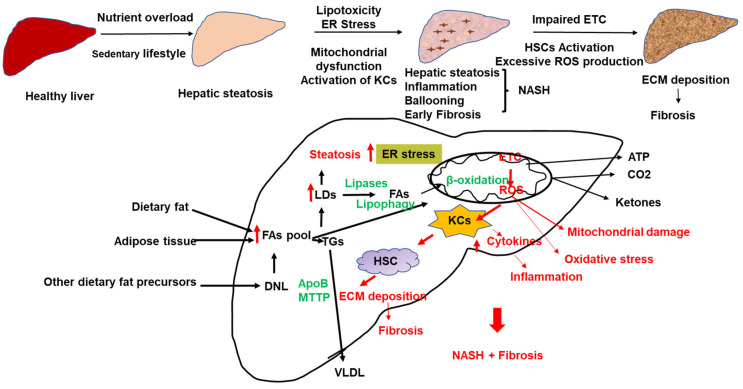
Pathogenic pathways involved in NAFLD. Top panel: schematic representing the progression of NAFLD, and the factors involved. Hepatic steatosis results from nutrient overload and a sedentary lifestyle. Multiple factors lead to inflammation and NASH, and the progression to fibrosis. Bottom panel: mechanisms for the development and the progression of NAFLD. The pool of FAs in the liver originates from dietary fat, adipose tissue lipolysis, or de novo lipogenesis (DNL) from carbohydrates or other dietary precursors. Inside the liver, FAs are (1) esterified into TG and assembled into very-low-density lipoprotein (VLDL) to be secreted in the circulation, (2) oxidized in the mitochondria (β-oxidation), or (3) stored in lipid droplets (LDs) (<5% of liver weight). LDs undergo lipid hydrolysis (via lipolysis and lipophagy) in fasting conditions to provide FAs for β-oxidation. With chronic nutrient overload and insulin resistance (increased adipose tissue lipolysis) in NAFLD, the input of FAs to the liver exceeds their disposal via VLDL secretion or β-oxidation. Lipotoxicity causes impaired LDs lipolysis and higher lipid accumulation in LDs (hepatic steatosis >5% of liver weight), leading to the endoplasmic reticulum (ER) stress, oxidative stress, and the activation of Kupffer (KCs) to produce inflammatory cytokines and inflammation. In addition, lipotoxicity causes mitochondrial dysfunction and impaired electron transport chain (ETC) function leading to ROS production. In a vicious cycle, ROS causes mitochondrial damage and aggravates NASH. Inflammation and ROS activate hepatic stellate cells (HSCs) to produce excessive extracellular matrix leading to progressive fibrosis.

The still-standing model for the development of inflammation and the progression of NAFLD is the multiple hits model that implicates multiple stressors [24,25] (Figure 1). While progress has been made in understanding the mechanisms underlying the development of hepatic steatosis, the pathogenesis of NASH is still not completely understood. Factors influencing NASH progression include lipotoxicity, endoplasmic reticulum (ER) stress, mitochondrial dysfunction, oxidative stress, gut endotoxins, and microbiota [19,25,26,27,28]. Lipid overload can induce lipotoxicity, inflammation, oxidative stress, and fibrosis (Figure 1). Western lifestyle, high calorie-rich diet, and decreased physical activity are among the most crucial factors in the development of NAFLD. Due to free FA overload, uncoupling respiration from ATP production might cause excessive ROS and MASH [28] (Figure 1).

The accumulation of fat in the liver and the development of NAFLD involves an imbalance between FA delivery to the liver (from the diet, de novo lipogenesis (DNL), and adipose tissue lipolysis), lipid synthesis and oxidation, and triglyceride (TG) export out of the liver in the form of very-low density lipoproteins (VLDLs) [29,30,31,32] (Figure 1). Both VLDL secretion and β-oxidation are elevated in the initial stages of NAFLD to compensate for increased FAs influx to the liver; however, a sustained influx of FAs to the liver leads to lipotoxicity, liver injury, and NASH [28,33]. NASH patients have lower VLDL secretion and lower FA oxidation than patients with fatty liver [28,33].

#### 2.2.1. Dietary Fatty Acids

Dietary FAs are absorbed from the small intestine, assembled into chylomicrons, and secreted into the blood, where the majority is stored in adipose tissue; the remaining are taken by the liver [34]. In the postprandial period, FAs in the liver are derived from chylomicron-derived spillover FAs and chylomicron remnants [35]. In the fasted state, FAs originated from adipose tissue lipolysis [35]. It has been estimated that, in patients with NAFLD, about 15% of liver FAs originate from the diet, 59% are derived from circulation, and 26% from DNL [36]. The FA composition of the diet may also influence hepatic fat accumulation [37].

#### 2.2.2. De novo-Lipogenesis

Insulin resistance drives de novo lipogenesis (DNL) in NAFLD [38,39]. Hepatic DNL is a highly regulated metabolic pathway by which the cells convert excess carbohydrates, commonly glucose, into FAs. Dietary glucose undergoes glycolysis and tricarboxylic acid (TCA) cycle to produce citrate in the mitochondria. The citrate is transported to the cytosol to generate acetyl-CoA by ATP-citrate lyase. Acetyl-CoA is converted to malonyl-CoA by acetyl-CoA carboxylases (ACC). In mammals, ACC is present in two major isoforms: ACC1 and ACC2. ACC1 is the main regulator of DNL in the liver. ACC1 is inactivated via phosphorylation by AMP-activated protein kinase (AMPK) [40]. In addition to phosphorylation, ACC1 is also inhibited by malonyl-CoA and palmitoyl-CoA [38,40,41] and activated by citrate [42]. Malonyl-CoA inhibits carnitine palmitoyl-CoA transferase 1 (CPT1), which regulates long-chain fatty acyl CoA import into the mitochondria for β-oxidation [43]. FA synthase (FASN) converts malonyl-CoA into palmitate, the first FA product in DNL. Palmitate undergoes elongation and desaturation reactions to generate complex FAs [44,45,46]. Multiple transcription factors control the expression of enzymes directly involved in DNL, including the sterol regulatory element-binding protein-1 (SREBP-1), carbohydrate responsive element-binding protein (ChREBP), and liver X receptors (LXRs) [47,48]. In addition to glucose, amino acids, short-chain FAs such as acetate and fructose also contribute to DNL.

#### 2.2.3. Fatty Acid Uptake

As mentioned above, 59% of FAs are derived from the circulation in NAFLD. The release of FAs from adipose tissue occurs under the control of adipose triglyceride lipase (ATGL), hormone-sensitive lipase, and monoglyceride lipase [49]. Insulin resistance in obesity and NAFLD increases adipose tissue lipolysis and the release of FAs in the circulation. The liver takes up FAs from the circulation through both passive diffusion and active transport. Different proteins take part in FA uptake in the liver, including the FA translocase CD36, the FA transport proteins (FATPs), and the FA binding proteins (FABPs). CD36 is closely associated with the development of NAFLD. CD36 expression is increased in animal models, and in humans with NAFLD [50,51,52,53]. CD36 knockout mice have normal rates of FA uptake compared to controls. However, upregulation of CD36 increases FA uptake in the liver, suggesting a role for the protein in pathogenic conditions [53,54,55,56,57,58]. Liver fat content in morbidly obese patients is associated with increased liver CD36 mRNA and protein levels [57]. The translocation of CD36 to the plasma membrane of the hepatocytes in NAFLD patients might be a key factor in the pathophysiology of hepatic steatosis [27]. The uptake of FAs by the liver drives hepatic steatosis and, when excessive, might cause lipotoxicity and contribute to the progression of NAFLD [27]. In addition to its role in FA uptake, CD36 might play other intracellular roles in lipid processing, such as VLDL secretion [50].

FATPs also play a role in the uptake of FAs in the liver. FATP2 and FATP5 are the two major FATPs present in the liver [59]. In mice, deletion of FATP2 or FATP5 decreased FA uptake in the liver [60,61]. Overexpression of FATP2 increases FA uptake in human hepatoma cells [58]. The level of FATP5 correlated inversely with histological features of MASH, including ballooning and fibrosis. Studies have shown that FATP5 expression is elevated in patients with less severe steatohepatitis but is reduced during advanced NASH [62].

Nine different FABPs have been identified with different tissue distributions. FABP1 is the highly expressed FABP in the liver and mediates the transport, storage, and use of FAs and their acyl-CoA derivatives; FABP1 may exert a protective effect against lipotoxicity by facilitating FAs oxidation or their incorporation into TGs [63]. Interestingly, FABP1 protein levels are upregulated in obese patients with steatosis, but it decreases in NASH with a further decrease in advanced fibrosis [63]. Other studies have shown no relationship between FABP1 expression and steatohepatitis histology [62].

The pool of FAs from the different pathways is then directed to LDs for storage as TGs, incorporated into lipoproteins and secreted into the circulation, used in β-oxidation, or used for posttranslational modifications.

#### 2.2.4. Triglyceride Synthesis

The glycerol-3-phosphate acyltransferase (GPAT) is the rate-limiting enzyme in the de novo pathway of TG synthesis. The glycerol-3-phosphate (G3P) pathway provides over 90% of the total TG synthesis. GPAT converts G3P and long-chain acyl-CoA to lysophosphatidic acid (LPA) [64]. In the endoplasmic reticulum, the acylglycer-ol-3-phosphate acyltransferases (AGPAT) acylates LPA to form phosphatidic acid (PA) [65]. PA is dephosphorylated by phosphatidate phosphohydrolase (PAP, Lipin) to form diacylglycerol (DG). The DG acyltransferase (DGAT) catalyzes the conversion of DG to TG [65,66]. Inhibition of DGAT2 in obese mice improved hepatic steatosis but aggravated liver damage and fibrosis [67], supporting the hypothesis that TGs have a protective role in the liver.

#### 2.2.5. Lipoprotein Secretion

Lipoproteins consist of a lipid core (TG and cholesteryl esters) surrounded by a monolayer composed of phospho-lipids, free cholesterol, and apolipoproteins. VLDL are TG-rich lipoproteins secreted by the liver and serve to transport FAs to peripheral organs such as adipose tissue, muscle, and the heart. Two apoprotein B are expressed in mammals: a long form (apoB100) in the human liver and a short form (apo48) in the intestine and rodent liver. The mRNA editing enzyme, apobec1, converts the cytidine at position 6666 of the full apoB mRNA to uracil, creating a premature stop codon in the intestine and the liver in rodents [68,69]. This short form of apoB mRNA is translated into an apoB48 protein, consisting of the N-terminal 48% of apoB100. The assembly of VLDL is conducted in two steps that involve apoB and mitochondrial transfer protein (MTTP) [70]. In the first step, apoB is co-translationally lipidated, in the ER, by MTTP to form a small primordial particle [70]. The mature VLDL particle is formed by further lipidation through fusion with LDs in the ER. VLDL secretion is linked to ER stress and NAFLD progression [71]. Although moderate exposure to FAs increases apoB100 secretion, prolonged exposure leads to ER stress, degradation of apoB, and decreased apoB secretion. The transport of VLDL from the ER to the Golgi appears to be mediated by specialized vesicles called VLDL transport vesicles containing Coat Protein II components such as the transmembrane 6 superfamily 2 (TM6SF2), the cargo receptor surfeit 4 (SURF4), the secretion associated Ras-related GTPase 1B (SAR1B) and meningioma-expressed antigen 6 (Mea6) [72,73]. Clinical and epidemiological studies have shown that TM6SF2 participates in the development of NAFLD. Liver-specific deletion of Tm6sf2 in mice shows steatosis and reduced VLDL TGs [74]. Newberry et al. recently reported that liver-specific deletion of TM6sf2 in mice impaired VLDL secretion and promoted hepatic steatosis, fibrosis, and (HCC) [75].

Defective VLDL assembly and secretion is one of the key contributing factors in the pathogenesis of NAFLD. Genetic defects in apoB (hypobetalipoproteinemia) and MTTP (abetalipoproteinemia) reduce VLDL secretion and hepatic steatosis. Rare apoB and MTTP mutations are associated with progressive liver disease [76,77]. Specific deletion of MTTP in the liver causes hepatic steatosis and complete inhibition of VLDL and apoB secretion [78,79]. Insulin reduces hepatic lipid export by inducing apoB100 degradation and suppressing MTTP synthesis [71]. In NAFLD, selective hepatic insulin resistance stimulates DNL without reducing VLDL production [80]. VLDL secretion is increased in patients with NAFLD, and liver triglyceride content is directly associated with VLDL-TG secretion rates [81]. However, when hepatic fat content exceeded 10%, VLDL TG secretion plateaued [82]. ApoB synthesis rates were lower in patients with NASH than in lean or obese controls without NASH [82]. Therefore, the ability of the liver to balance lipid storage and VLDL secretion is critical in determining the NAFLD outcome (Figure 1).

#### 2.2.6. Lipid Droplets Formation and Lipophagy

The accumulation of TG in LDs results from increased TG synthesis and LDs formation or decreased LDs catabolism. Under physiological conditions, the liver stores less than 5% of lipids in the form of TG in cytoplasmic LDs. With nutrient overload and obesity, alteration of hepatic metabolism leads to a higher accumulation of LDs in the liver and NAFLD [29]. While the degree of steatosis determines the susceptibility of the liver to steatohepatitis, hepatic TG accumulation is liver-protective [83,84]. It is now accepted that LDs are dynamic organelles involved in many cellular processes beyond lipid storage [85]. The release of FAs from TGs is regulated by cytosolic lipases, particularly ATGL, or through autophagy of LDs, lipophagy. ATGL has been shown to function as an inducer of autophagy/lipophagy [86]. Deletion of ATGL in mice or knockdown using short hairpin RNA promoted hepatic steatosis [87,88], while overexpression of ATGL in the liver alleviated steatosis [89]. Pharmacological inhibition of ATGL in HFD-fed mice lowered hepatocellular FA levels. It showed less severe liver damage than mice fed HFD without treatment [90], consistent with the hypothesis that storage of TG in LDs is liver protective. The release of FAs from LDs regulates the peroxisome proliferator-activated receptor-α (PPARα)-mediated β-oxidation. ATGL regulates mitochondrial biogenesis and the expression of β-oxidation genes through its regulation of SIRT1 [90]. Chaperone-mediated autophagy has been shown to contribute to hepatic LD catabolism via its degradation of the LD protein perilipin 2 (PLIN2) [91]. The degradation of this proteins allows ATGL to gain access to LDs and facilitate lipolysis. Studies in liver-specific SIRT1 knockout mice showed that SIRT1 is needed for ATGL-mediated induction of autophagy and lipophagy [86]. ATGL appears to channel the released FAs to oxidative pathways selectively; it does not influence VLDL secretion [92,93]. Loss of ATGL or CPT 2 resulted in hepatic steatosis. However, the loss of both components resulted in significant inflammation and fibrosis [94]. Therefore, ATGL is essential for providing the substrate for FA oxidation and coordinating the transcriptional program for FA oxidation.

Lipophagy is a specific form of autophagy where LDs are engulfed by the autophagosomes and then degraded in the lysosomes [72,95,96,97]. Inhibition of lipophagy in the liver promotes LDs accumulation and attenuates β-oxidation of the released FAs [92,95]. A recent study has provided mechanistic insights into the potential crosstalk between ATGL-catalyzed lipolysis and autophagy/lipophagy in the liver. This study showed that ATGL has an LC3-interacting region that facilitates its interaction with LC3-containing organelles [98]. Mutating the LC3-interacting region prevented ATGL from targeting LDs, suggesting important crosstalk between autophagy and ATGL. Additionally, PNPLA5, a putative neutral lipid hydrolase and member of the patatin-like phospho-lipase-domain-containing family, which includes ATGL/PNPLA2, promotes autophagy, suggesting that cytosolic lipases could act upstream to regulate autophagy/lipophagy [99]. While ATGL loss was associated with large LDs, the accumulation of small LDs has been suggested to arise from a defect in lipophagy [100]. LDs biology and macroautophagy have been recently reviewed by Filali-Moncef [101].

#### 2.2.7. Role of the Mitochondria in NAFLD

The liver has between 500 and 4000 mitochondria/hepatocytes that occupy 18% of the cell volume [102]. In addition to the β-oxidation and the production of ATP, mitochondria hold other essential functions, including the generation of ROS and Ca2+ signaling [103,104,105]. More recently, mitochondria have been linked to the activation of the inflammasome and cell apoptosis [106]. In addition, mitochondria interact with other cell organelles such as the ER, LDs, and lysosomes [107,108,109]. Therefore, mitochondrial dysfunction has been implicated in many metabolic diseases, including NAFLD, NASH, and HCC.

NAFLD results from excessive exposure of mitochondria to nutrients compared to their ATP demand. Nutrient availability regulates mitochondrial function, cellular ATP demand, redox signaling, and the response of cellular processes supporting mitochondrial health [110]. In addition to the mechanisms for the pathophysiology of NAFLD described above, accumulating evidence suggests that changes in mitochondrial function may be a major contributing factor [31,111,112]. Multiple mitochondria-associated factors contribute to the development and progression of NAFLD, including reduced β-oxidation, impaired ETC and ATP depletion, over-production of ROS, oxidative stress-mediated cell damage, and ultra-structural mitochondrial changes. These changes in mitochondrial function and structure aggravate hepatic lipid accumulation and trigger inflammation and fibrogenesis, therefore, contributing to the development and progression of NAFLD [31,111,112,113]. In humans and mice, mitochondria appear to adapt to substrate overload at the initial stages of NAFLD by increasing β-oxidation, mitochondrial respiration, and ketogenesis [112,113]. However, this adaptation is lost during the progression to NASH, associated with mitochondrial dysfunction (incomplete β-oxidation, impaired ketogenesis, reduced ATP production, and leakage in the ETC) [111]. Obese individuals without MASH have increased ETC activity compared to lean subjects, suggesting hepatic mitochondrial adaptation in the first stages of obesity-related insulin resistance [28]. This loss of mitochondrial adaptation results in increased ROS production is associated with mDNA damage, ER stress, inflammation, and eventual cell death. Mitochondrial damage, in turn, causes more ROS production. Koliaki et al. showed that NASH patients showed an additional increase in mitochondrial ROS production and a decrease in their oxidative function compared to patients with simple steatosis [28]. This added increase in mitochondrial ROS in NASH was concomitant with a decrease in catalase activity [28]. ROS activates the inflammatory signaling pathways such as NF-κB and JNK pathways and increases the expressions of inflammatory cytokines such as TNF-α and TGF-β [28]. Inflammation leads to the transformation of hepatic stellate cells into collagen, secreting myofibroblasts and liver fibrosis. In addition, damaged mDNA activates the inflammasome [114]. Furthermore, mitochondrial structural changes were found in NASH, in addition to functional abnormalities. NASH is associated with loss of mitochondrial cristae, para-crystalline inclusions, and mitochondria swelling [115,116,117].

##### Structure of the Mitochondria

Mitochondria are cell organelles with a complex structure that includes an outer mitochondrial membrane (OMM), inner mitochondrial membrane (IMM), and a mitochondrial matrix. Most mitochondrial proteins are made in the cytoplasm and transported to the mitochondria. The OMM is porous and allows the passage of ions and small un-charged molecules. The IMM is complex and consists of the complexes of the electron transport system, the ATP synthase, and transport proteins [118]. The mitochondrial matrix holds the mDNA with thirteen structural genes that encode subunits essential for respiratory complexes I, III, IV, and V of the mitochondrial ETC generating ATP. The matrix also holds enzymes involved in the oxidation of FAs and pyruvate in the TCA cycle.

##### Fatty Acid Oxidation and ATP Production

Mitochondria are the main site for energy production, in the form of ATP, via the metabolism of pyruvate and FAs. To undergo β-oxidation, FAs in the cytosol need to enter the mitochondria. While short- and medium-chain FAs diffuse freely into the mitochondrial matrix. Long-chain FAs are activated to acyl-coenzyme A (acyl-CoA) by long-chain acyl-CoA synthase in the OMM. At the outer side of IMM, CPT1 transfers acyl groups from acyl-CoA to carnitine to form acylcarnitine. The carrier protein carnitine-acylcarnitine translocase mediates the transport of the acylcarnitine molecules across IMM. CPT2, in the mitochondrial matrix, converts acylcarnitine to carnitine and acyl-CoA. Inside the mitochondria, acyl-CoA is degraded via cycles of β-oxidation consisting of four enzymatic steps [119]. Each cycle shortens the acyl-CoA by liberating the two carboxy-terminal carbon atoms as acetyl-CoA. The first step of β-oxidation consists of the dehydrogenation of the acyl-CoA to trans-2-enoyl-CoA by an acyl-CoA dehydrogenase. The mitochondrial trifunctional protein complex (MTP) catalyzes the last three steps. In the second step, hydration catalyzed by an enoyl-CoA hydratase generates (S)-3-hydroxyacyl-CoA, which is subsequently dehydrogenated (in the third step) to 3-ketoacyl-CoA in a reaction performed by (S)-3-hydroxyacyl-CoA dehydrogenase [31]. In the last step, a thiolase cleaves the 3-ketoacyl-CoA to generate a two-carbon chain–shortened acyl-CoA and an acetyl-CoA. In addition to acetyl-CoA feeding into the TCA cycle and ketogenesis, β-oxidation produces nicotinamide adenine dinucleotide (NADH) and flavin adenine dinucleotide (FADH2). The ETC uses the NADH and FADH2 produced by β-oxidation and the TCA cycle to produce ATP. ATP is produced by oxidative phosphorylation, which pairs oxidation of NADH or FADH2 and phosphorylation of ADP to form ATP [119].

##### Production of Reactive Oxygen Species

Mitochondria are the main site for ROS production (90% of cellular ROS). A fraction of electrons may leak from the ETC during ATP production and react with oxygen to form ROS. In normal conditions, about 1–2% of mitochondrial oxygen consumption results in ROS production [120]. In these conditions, ROS acts as signaling molecules and non-enzymatic and enzymatic antioxidant mechanisms counter its unnecessary generation. Non-enzymatic antioxidant molecules such as ascorbic acid (vitamin C), α-tocopherol (vitamin E), glutathione (GSH), carotenoids, and flavonoids remove free radicals from the body. The antioxidant enzymes include superoxide dismutase (SOD), glutathione peroxidase (GPx), and catalase (CAT). Uncontrolled mitochondrial oxidative stress interrupts ATP production in the mitochondria. In NAFLD, sustained free FAs flux and chronic production of acetyl CoA can uncouple the TCA cycle function from the mitochondrial respiration leading to increased ROS generation [121] (Figure 1). Excessive ROS production causes hepatocellular oxidative damage and the progression of NAFLD [112,121] (Figure 1). ROS release outside the hepatocytes contributes to HSCs activation and extracellular matrix deposition (Figure 1). In addition, excess free FAs in the liver can promote the accumulation of toxic lipid intermediates, such as ceramides [122]. Studies have shown that using antioxidants to remove ROS in the mitochondrial matrix and cytosol protects from simple steatosis and NASH [123]. Hepatocyte-specific deletion of GPX1 protected mice from diet-induced NASH [123]. However, the limited studies on humans often show conflicting results [124,125,126,127,128]. Indeed, elevated ROS production was associated with an increase in detoxification and antioxidant capacity in hepatic steatosis but not in NASH, indicating that mechanisms to cope with excess ROS generation may be insufficient in NASH [28].

##### Mitochondria Quality Control

Excessive hepatocellular lipids simultaneously stimulate mitochondrial FA oxidation and the production of ROS. In a vicious cycle, damaged mitochondria become dysfunctional, causing impaired oxidative phosphorylation, and increased production of ROS. Excessive and uncontrolled production of ROS in the mitochondria damages mitochondrial components, including membranes, proteins, and mDNA, and triggers the mitochondrial quality control (MQC) [129,130,131]. MQC includes biogenesis, fission, fusion, and mitophagy. As a first response to stress/ROS, mitochondria first attempt to maintain their structure and function through the antioxidants, DNA repair, protein folding, and degradation processes. Mitochondrial biogenesis, fusion, and fission compensate for mitochondrial function. A broader MQC system is initiated if this first response is ineffective [132,133]. Damaged mitochondria can be repaired by fusion with other healthy mitochondria, but severely damaged mitochondria are separated from healthy ones through fission and then targeted for degradation by mitophagy [134,135]. Failure of the MQC processes results in mitochondrial dysfunction and is one of the underlying causes of NAFLD progression [136].

Mitophagy is the autophagosome’s selective elimination of dysfunctional or de trop mitochondria. The recognition of damaged mitochondria by the autophagosome occurs through LC3 adapters in a ubiquitin-dependent and independent pathway. PTEN-induced putative kinase 1 (PINK1)/Parkin pathway is the most studied pathway for mitophagy. PINK1 is degraded by matrix processing peptidase (MPP) and the presenilin-associated rhomboid protease-like (PARL) in healthy mitochondria [135]. On damaged mitochondria, PINK1 accumulates and signals impaired mitochondria to parkin for elimination [135]. The ubiquitin kinase PINK1 phosphorylates ubiquitin to activate the ubiquitin ligase parkin [135,137]. The mitochondrial outer membrane GTPase mitofusin (Mfn)2 has been proposed to mediate Parkin recruitment to damaged mitochondria. PINK1 phosphorylates Mfn2 and promotes its parkin-mediated ubiquitination [138]. Parkin ubiquitinates proteins of the OMM and promotes their interaction with mitophagy adaptors such as P62, NBR1, and HDAC6 [137]. These adapters have an LC3-interacting region (LIR) motif that LC3 recognizes and recruits the tagged mitochondria to the autophagosome [137]. In the mitophagy receptor pathway, receptors such as Nip3-like protein X (NIX) and BCL2/Adenovirus E1B 19 kDa, interacting protein 3 (Bnip3) found on the mitochondria directly bind to LC3 to induce mitochondrial engulfment by an autophagosome for the elimination of damaged mitochondria by the lysosomes. Bnip3 null mice show increased ROS and inflammation associated with lower mitochondrial membrane potential, abnormal structure, and reduced oxygen consumption [139].

Impaired mitophagy has been implicated in a broad spectrum of human diseases [140]. Deregulation of mitophagy impairs the synthesis of new healthy mitochondria and leads to the accumulation of defective mitochondria. In addition, mitophagy plays a role activating of the inflammasome [106]. Moore et al. have recently linked compromised mitochondria to increasing NAFLD severity in patients with obesity [141]. They reported a 40–50% reduction in β-oxidation in NASH patients, associated with increased hepatic ROS production and a reduction in markers of mitochondrial biogenesis, autophagy, mitophagy, fission, and fusion [141]. Mitophagy is subjected to hormonal regulation. Thyroid hormones have been shown to alleviate NAFLD through increased FA oxidation and stimulation of mitophagy and mitochondrial biogenesis [142,143]. Thyroid hormones upregulate the expression of BNIP3, NIX, ULK1, p62, and LC3 mRNA [144]. The coordination between mitochondrial biogenesis and mitophagy fine-tunes the quantity and quality of the mitochondrial pool, allowing cells to adjust their mitochondrial content in response to cellular metabolic state, stress, and other intracellular environmental and hormonal signals.

##### ER-Mitochondria Contacts

The lipid composition of the mitochondrial membranes is critical for mitochondrial architecture and functional integrity. The conservation of normal mitochondrial lipid composition depends on the mitochondria to synthesize phospholipids and the traffic of lipids from the ER to mitochondria [145,146]. Mitochondria-associated ER membranes (MAMs) are key sites for the synthesis and traffic of phospholipids [147]. Phosphatidylserine (PS) is synthesized in the ER and is transferred into mitochondria by transient membrane contact between MAMs and the OMM. In the mitochondria, PS is converted into phosphatidylethanolamine, then transferred to the ER to be transformed into phosphatidylcholine. Studies provided evidence to show that mfn2 ties the ER to the mitochondria, an interaction needed for efficient mitochondrial calcium uptake and mitophagy [148]. Embryonic fibroblast cells from mfn2 knockout mice show decreased ER-mitochondria contacts suggesting that mfn2 acts as an ER-mitochondria tether involved in calcium signaling [148]. As mentioned above, mnf2 is an essential regulator of the MQC, mitochondrial morphology, and inflammasome [138]. MAMs have been proposed as nutrient, stress, and immune sensors that might link nutrient sensing to mitochondrial flexibility [149,150]. The ER-mitochondria communication might contribute to the metabolic inflexibility and the proinflammatory status associated with metabolic diseases such as NAFLD [149].

## 3. NAFLD Treatments

Despite its epidemic proportions, no pharmacological treatments are currently approved to treat NAFLD specifically. Pharmacological anti-diabetic, anti-lipidemic, and natural bile acid treatments have been used to treat NAFLD but have drawbacks [151,152]. Lifestyle interventions, diet, and exercise are currently the recommended interventions for managing NAFLD. This section will focus on the current intervention for the management of NAFLD and the SIRTs as potential contenders for NAFLD treatment.

### 3.1. Current Interventions in the Management of NAFLD

#### 3.1.1. Lifestyle Interventions

Lifestyle interventions have positively impacted NAFLD even in the absence of weight loss [153,154,155]. It is now recognized that a 5% weight reduction is associated with reduced liver fat and improved liver injury while body weight loss of >7% body weight improved NASH based on histology [153,156,157,158,159]. Importantly, weight loss percentage correlated with improved NASH histologic parameters [157]. While inflammation is essential for disease progression, the strongest predictor of mortality in patients with NASH is hepatic fibrosis [8,160]. Among patients who lost ~10% body weight, 90% showed improved NASH, and about ~45% showed fibrosis regression [160,161]. Lifestyle interventions involving a combination of calorie restriction and exercise have a higher impact on reducing liver fat [161]. However, more than 50% of patients included in clinical trials could not achieve this weight loss level [162]. Therefore, even though lifestyle interventions positively impacted NAFLD, sustained lifestyle changes are difficult to achieve [163].

Dietary interventions improve NAFLD with or without physical activity; however, the composition of the diet and the dietary pattern is still a matter of debate [157,159,162,164]. The picture is a little clearer in exercise interventions, as there is agreement that, in most clinical and preclinical studies, all exercise modalities and intensities are beneficial in NAFLD. Exercise has been shown to decrease hepatic steatosis, liver enzymes, blood glucose, and insulin and improve lipid profile, with or without dietary interventions [154,157,161,165]. Even without weight loss, regular exercise reduced hepatic lipids [166,167]. Exercise modulates FA synthesis and oxidation, mitochondria bioenergetics, and structure in the liver, thus preventing liver damage [166,168,169,170]. Exercise reduces hepatic fat by 1) down-regulating FA synthesis and upregulating FA oxidation, 2) decreasing oxidative stress and increasing antioxidant enzymes, and 3) reducing inflammation [171,172]. However, further research is needed to understand the underpinning mechanisms involved in the role of mitochondria and the epigenetics/posttranslational related mechanisms involved in the liver adaptation to lifestyle interventions. The 2018 ASSLD practice guidance states that weight loss reduces hepatic steatosis, achieved with hypocaloric diet, increased physical activity, or both. A combination of hypocaloric diet and moderate-intensity exercise is likely to result in sustained weight loss over time. Body weight loss of 3–5% improves steatosis, 7–10% body weight loss is needed to improve and including fibrosis [173].

#### 3.1.2. Bariatric Surgery

Bariatric surgery is now recommended as an effective approach to treating clinically severe obesity and its complications [174,175]. Based on liver biopsy, bariatric surgery in morbidly obese patients improves steatosis, NASH, and liver fibrosis in 30% of patients, [176]. However, despite the significant benefit of bariatric surgery for resolving NASH, the risk of these surgeries currently excludes their use as first-line therapy for NAFL and NASH patients.

#### 3.1.3. Pharmaceutical Therapies

As of now, no pharmacological treatment is approved to treat NAFLD. Better understanding of the pathogenesis of NAFLD has led to the investigation of potential drug molecules in clinical trials, to determine their efficacy and safety in the resolution of steatohepatitis and fibrosis. These drug molecules target several aspects of metabolic disruption including lipotoxicity, oxidative stress, mitochondrial dysfunction, and fibrosis. The drugs currently used for the treatment of NAFLD include off label treatments such antioxidant (VitE) and antidiabetic drugs agents such as pioglitazone. The farnesoid X receptor agonist obeticholic acid (OCA), the thyroid hormone receptor THRβ agonist (Resmetirom), and Aramchol (bile acid and and FA conjugate, cholic acid–arachidic acid), are drugs currently in phase 3 randomized clinical trials (RCTs) for the treatment of noncirrhotic NASH [177]. Drugs in phase 3 and several drugs in phase II phase 2 RCTs are reviewed in this section.

##### Vitamin E

Vitamin E in the bioactive α-tocopherol is a potent antioxidant that helps maintain intracellular redox status by counteracting the increase in oxidative stress. In the PIVENS, phase 3 RCT, non-diabetic participants with NASH based on liver biopsy, were administered daily treatments of 30 mg Pioglitazone, 800 IU VitE, or placebo for 96 weeks. Vitamin E therapy, as compared with placebo improved NASH. Both VitE and Pioglitazone significantly lowered ALT and AST levels, reduced hepatic steatosis and lobular inflammation but without improvement in fibrosis scores. The side effects were weight gain with Pioglitazone (ClinicalTrials.gov number, NCT00063622) [178]. In the TONIC trial, VitE (800 IU/day) or metformin 500 mg twice-daily were tested against placebo in children with biopsy-proven NAFLD [179]. VitE improved NASH compared to placebo [179]. The 2018 AASLD updated practice guidance stated that VitE administered at a of 800 IU/day may be considered for this patient population. However, VitE is not recommended to treat NASH in diabetic patients, NAFLD without liver biopsy, NASH cirrhosis, or cryptogenic cirrhosis [173]. Long-term safety of high-dose vitamin E need further evaluation for efficacy and safety. Metformin is not recommended for treating NASH in adult patients [173].

##### Pioglitazone, PPAR Agonist

PPAR targeting drugs, including thiazolidinediones such as Pioglitazone, are clinically used to treat T2D. In the PIVENS trial, treatment of nondiabetic NASH subjects with Pioglitazone (30mg/day) reduced hepatic steatosis, lobular inflammation, hepatocellular ballooning, improved insulin resistance and liver-enzyme levels, and reduced liver injury [178]. However, Pioglitazone treatment was associated with increased body weight gain [178]. Pioglitazone is contraindicated in patients with established heart failure or with an increased risk of heart failure [180]. Low dose of Pioglitazone (15mg/day) is being evaluated in a phase II clinical trial (Clinicaltrials.gov, NCT04501406) to evaluate the effect Pioglitazone on liver histology in patients with NASH. Clinical trials evaluating other PPAR agonists are ongoing [181]. The 2018 AASLD practice guidance states that Pioglitazone may be used to treat patients with and without T2D, with biopsy-proven NASH. Further pioglitazone efficacy and safety evaluation is needed. The 2018 ASSLD practice guidance states that Pioglitazone should not be used to treat NAFLD without biopsy-proven NASH [173].

##### Obeticholic Acid, Farnesoid X Receptor

Farnesoid X receptor FXR is a ligand-activated nuclear receptor transcription factor abundantly expressed in the liver, intestine, and kidney [182]. FXR regulates several metabolic functions, including bile acid synthesis, glucose homeostasis, and lipid metabolism [183]. Activation of FXR by ligands induces a small heterodimer partner, which suppresses CYP7A1 gene expression. CYP7A1 is a rate-limiting enzyme that converts cholesterol to bile acids, inhibition of which results in the reduced rate of bile synthesis in the liver [182]. Bile acids are FXR natural ligands. Activation of FXR in both hepatocytes and enterocytes reduced bile acid synthesis and improved hepatic steatosis and inflammation [183]. In preclinical studies, FXR agonists have shown beneficial effect on NASH [183]. In the FLINT study [184], FXR ligand obeticholic acid (OCA) was evaluated for the treatment of NASH (clinicaltrail.gov, NCT01265498). OCA improved NASH, fibrosis, and markers of hepatic damage [184]. REGENERATE is a phase 3 global RCT to evaluate the impact of OCA on NASH with fibrosis (clinicaltrials.gov, NCT02548351). Histologic assessment of patients with NASH and F2-F3 fibrosis demonstrated significant improvement in fibrosis by one stage with no resolution of NASH. The most adverse effect of OCA treatment are high rates of pruritus [185].

##### Resmetirom, Thyroid Hormone Receptor THRβ Agonists

Thyroid hormones (THs) regulate many processes involved in hepatic TG and cholesterol metabolism. Triiodothyronine (T3) is the major active form of THs and exerts its action by binding to two TH receptor, THRα and THRβ, which act as ligand-inducible transcription factors. THs increases influx of FAs in the liver by upregulating the expression of CD36, FABP and lipogenic genes [186]. However, THs increases TAG hydrolysis by stimulating the transcription and activities of ATGL, therefore mediating the mobilization of free FAs from TG stores and their subsequent β-oxidation [186]. THs also downregulate stearoyl-CoA desaturase-1 (SCD1), a key enzyme involved in triglyceride biosynthesis and GPAT, consequently limiting the storage of LDRs in the liver [186]. THRβ is expressed in the liver while THRα is expressed in the heart and bone. Resmetirom is a selective THRβ agonist that has been developed to specifically activate THRβ in the liver and eliminate the side effects associated with the activation of THs in other tissues. Resmetirom was evaluated in adults with NASH (clinicaltrials.gov, NCT02912260) [187]. Compared to patients treated with placebo, Resmetirom reduced liver fat on MRI-PDFF, and reduced NASH [187]. Resmetirom treatment appears to be safe, the adverse effects are mild diarrhea and nausea. Resmetirom is being evaluated in phase III MAESTRO-NASH trial to assess its efficacy and safety of in patients with NASH and fibrosis (clinical trial.gov, NCT03900429).

##### Cholic Acid-Arachidic Acid Conjugate

Aramchol is a synthetic conjugate of bile acid (cholic acid) and FA (arachidic acid) that inhibits SCD-1. Treatment of methionine and choline deficient (MCD) mouse, a mouse model of NASH, with Aramchol improved NASH and fibrosis [188]. In the phase II 2b RCT, Aramchol was evaluated for its efficacy and safety versus placebo, in patients with NASH who had overweight or obesity and had confirmed prediabetes or T2DM (ARREST, clinical trial NCT02279524). Aramchol (daily doses of 400 mg or 600 mg) for 52 weeks decreased liver fat and improved liver enzymes with a trend toward higher NASH improvement with the 600 mg dose [189]. Aramchol is currently being investigated in the ARMOR phase III clinical trial (clinical trial.gov, NCT04104321) to evaluate its efficacy and safety in patients with NASH and F2-F3 fibrosis.

##### Glucagon-Like Peptide Receptor Agonist

Glucagon-like peptide (GLP-1) and glucose-dependent insulinotropic peptide (GIP) are the two primary incretin hormones secreted by the L-cells and K-cells of the small intestine, respectively [190]. GIP and GLP-1 bind to their specific G-protein coupled receptors to initiate downstream signaling events in pancreatic β cells to stimulate glucose-dependent insulin secretion [190]. In a phase II study in patients with NASH and fibrosis, the GLP-1 receptor Semaglutide, improved NASH without worsening of fibrosis [191]. In the LEAN clinical trial, the GLP-1 receptor agonist Liraglutide, showed efficacy in reducing liver fat content as well as liver enzymes in patients with NASH (clinical trial.gov, NCT01237119). Liraglutide was safe, well tolerated, and led to histological resolution of NASH [192]. Liraglutide was associated with greater weight loss but also gastrointestinal side effects [192]. The SYNERGY-NASH, a phase II study to assess Tirzepatide, a dual GIP/GLP-1 receptor agonist in participants with NASH (clinicaltrials.gov, NCT04166773) is underway (NCT04166773). Phase II clinical trials using other agonists, or a combination of agonists are currently ongoing. The 2018 ASSLD practice guidance states GLP-1 agonists are not currently considered to specifically treat liver disease in patients with NAFLD or NASH [173].

##### Sodium-Glucose Co-Transporter Type 2 Inhibitor

Sodium-glucose co-transporter type 2 inhibitors (SGLT2i) are used as antidiabetic drugs. SGLT2 is almost exclusively expressed in the kidney and reabsorbs >90% of the glucose filtered at the glomerulus [193]. Pharmacological inhibition of SGLT2 using Dapagliflozin improved liver steatosis and attenuated liver fibrosis only in patients with significant liver fibrosis [194]. In patients with T2D and NAFLD, Dapagliflozin improved liver function parameters and decreased serum level of hepatocytes-secreted soluble dipeptidyl peptidase-4 (DPP4) which is responsible for adipose tissue inflammation and insulin resistance [194]. A phase III multi-center RCT is ongoing to assess the safety and the efficacy of Dapagliflozin in improving NASH (clinicaltrial.gov, NCT03723252). Other SGLT2i are being evaluated in phase II clinical trials.

##### Fibroblast Growth Factors Activators

Fibroblast growth factors (FGFs) are a superfamily of metabolic hormones that regulate many aspects of the whole-body health. Circulating FGF21 is liver-derived [195], but it is also expressed in several other tissues, such as the pancreas, muscle, and adipose. FGF19 and its mouse ortholog FGF15 [195] are gut-produced hormones with the highest expression in the ileum [195]. Both FGF19 and FGF21 play an important role in the liver [195,196]. FGF19 and FGF21 signal through widely FGF receptors in the body. The activity of FGF19 and FG21 requires a transmembrane scaffold protein bKlotho (KLB) [195]. FGF21 analogs have demonstrated efficacy in animal models and humans with NASH, and several clinical trials with FGF21 analogs are currently underway. Two FGF21 molecule in RCTs are reviewed here, Pegbefermin and Efruxifermin.

##### Pegbelfermin, FGF21 Analog

Pegbelfermin, a PEGylated, recombinant human FGF21 analog, was evaluated for its efficacy and safety in a phase II RCT. Subcutaneous treatment with Pegbelfermin, in obese/overweight subjects with NASH, for 16 weeks reduced liver fat measured by MRI-PDFF, improved biomarkers of metabolic function (adiponectin and lipid concentrations), and biomarkers of fibrosis. Pegbelfermin is being evaluated for its efficacy and safety in a two phase 2b RCT in patients with NASH and stage 3 fibrosis FALCON 1 (clinicaltrials.gov, NCT03486899), or in cirrhosis, FALCON 2 (clinicaltrials.gov, NCT03486912).

##### Efruxifermin, Fc-FGF21 Fusion Protein

BALANCED, a Phase 2a RCT in patients with histologically confirmed NASH, evaluated the safety and efficacy of fruxifermin, a long-acting Fc-FGF21 fusion protein, in a 16-week study (clinicaltrials.gov NCT03976401) [197]. The treatment with Efruxifermin was safe, except for diarrhea and nausea in approximately 30% of participants. Efruxifermin improved NAFLD activity score (NAS) and fibrosis, reduced body weight and liver fat content, and improved circulating TG and increased HDL.

##### mRNA Encoding Human FGF21

The therapeutic levels of FGF21 were achieved following subcutaneous administration of mRNA encoding human FGF21 proteins. FGF21 mRNA was assessed following 2-weeks repeated subcutaneous injections in diet-induced obese mice, which resulted in marked decreases in body weight, plasma insulin levels, and hepatic steatosis [198]. Studies in both lean and diet induced obesity mice showed that mRNA encoding human proteins provided better therapeutic coverage than recombinant proteins, in vivo suggesting that FGF 21 mRNA therapy might have the potential to treat T2D and NASH.

##### Statins

T2D is associated with increased risk of both cardiovascular disease (CVD) NASH and liver fibrosis. Satin is known to lower CVD. The effect of statins on liver fibrosis and hepatic steatosis were assessed in adult patients with T2D, in a cross-sectional study using data from the 2017–2018 cycle of the National Health and Nutrition Examination Survey (NHANES). Statin use was associated with lower odds of advanced fibrosis [199]. The updated ASSLD practice guidance indicates that statins can be used in the treatment of dyslipidemia in patients with NAFLD and NASH. While statins may be used in patients with NASH cirrhosis, they should be avoided in patients with decompensated cirrhosis [173]. Studies evaluating the safety and efficacy of statins in NASH and fibrosis are limited. STAT-NASH, a phase 2 RCT, is underway to assess the treatment of NASH with statins (clinicaltrials.gov, NCT04679376).

### 3.2. Sirtuins as Targets for NAFLD Treatment

**Figure 2:**
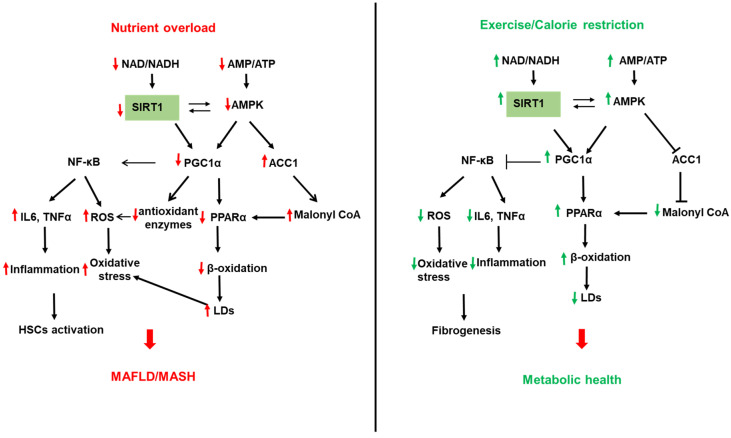
Role of sirtuin (SIRT) 1 in NAFLD. Left panel: Reduced SIRT1 with nutrient overload leads to the acetylation and inactivation of the peroxisome proliferator-activated receptor (PPAR) gamma-coactivator α (PGC1α). Reduced PGC1α activity downregulates β-oxidation by reducing PPARα expression and decreasing mitochondrial biogenesis, leading to increased lipid accumulation in LDs and hepatic steatosis. In addition, reduced SIRT1 activity decreases the expression of the antioxidant enzymes (SOD2 and catalase), leading to increased cellular ROS. On the other hand, reduced PGC1α activity increases the expression of the transcription factor NF-κB to increase inflammatory cytokines such as TNFα and IL6 and ROS generation. In addition, reduced AMP/ATP ratio with nutrient overload inactivates AMP-activated protein kinase (AMPK), leading to reduced NAD/ATP ratio and reduced SIRT1 and PGC1α activities. AMPK also directly regulates PGC1 via modulation of its phosphorylation. Reduced AMPK also reduces ACC1 phosphorylation and increases its activity leading to increased malonyl CoA and inhibition of β-oxidation. Right panel: Upregulation of SIRT1 by exercise, calorie restriction, and fasting upregulates SIRT1 leading to a healthier liver via opposite effects on B-oxidation, inflammation, and ROS generation than chronic nutrient overload.

**Figure 3:**
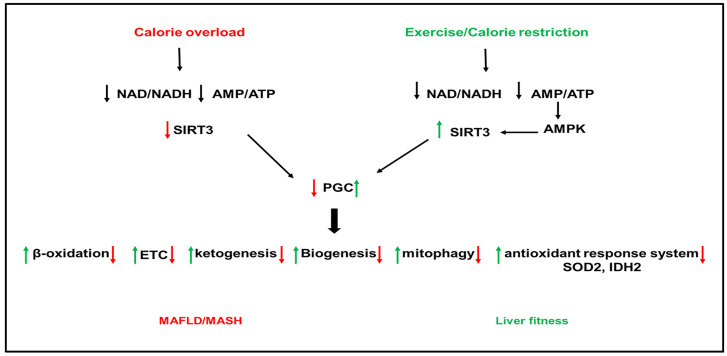
Role of sirtuins (SIRT) 3 in NAFLD. Calorie overload and liver lipotoxicity downregulate SIRT3 activity leading to the inactivation of PGC1α. The inactivation of PGC1α downregulates the downstream pathways, including β-oxidation, antioxidant enzymes (superoxide dismutase (SOD)2 and isocitrate dehydrogenase (IDAH)2, the ETC, ketogenesis, biogenesis, and mitophagy, leading to the development of hepatic steatosis, inflammation, and fibrogenesis. Activation of SIRT3 by exercise and calorie restriction activates PGC1α with opposite effects on PGC1 downstream pathways than nutrient overload and lipotoxicity, leading to metabolic health.

SIRTs represent potential targets for the treatment of NAFLD due to their role in hepatic lipid and carbohydrate metabolism, insulin signaling, redox signaling, and inflammation [200,201,202,203,204,205,206]. SIRTs are a family of seven members (SIRT1–7) with different cellular localization and are implicated in multiple cellular processes. SIRT1 and SIRT3 are NAD+-dependent deacetylases regulated by cellular NAD^+^/NADH ratio. SIRT1 and SIRT3 are upregulated by fasting, calorie restriction, exercise, and polyphenols and downregulated by nutrient overload. This section focuses on the most studied SIRTs, SIRT1 and SIRT3 (Figure 2 and Figure 3).

#### 3.2.1. SIRT1 and NAFLD

SIRT1 is found in the nucleus and shuttles between the nucleus and cytoplasm under physiological and pathological conditions [206,207]. SIRT1 regulates, via deacetylation of transcription factors and proteins, multiple metabolic pathways in the liver, including FA synthesis and oxidation, oxidative phosphorylation, inflammation, mitochondrial biogenesis, and autophagy [202,206,208,209] (Figure 2). The involvement of SIRTs in NAFLD has been shown in both human and animal models of NAFLD. SIRT1 is downregulated in humans with NAFLD, and this was associated with increased expression of lipogenic proteins, such as SREBP1, ACC, and FAS [210]. Furthermore, the lack of SIRT1 catalytic activity promoted the release of free FAs from mesenteric adipose tissue and aggravated NAFLD [211]. SIRT1 levels were low in obese compared to lean patients and lower in obese patients with severe hepatic steatosis compared to obese patients with mild hepatic steatosis [212]. PPARγ coactivator-α (PGC1α) directly coactivates multiple transcription factors, including nuclear receptors such as PPARs, the thyroid hormone receptor, estrogen receptors, and estrogen-related receptors (ERRs). In addition, PGC1α coactivates transcription factors such as the family of forkhead O-box (FOXO) transcription factors [213]. PGC1α activity is regulated by expression levels and posttranslational modifications such as acetylation and phosphorylation. SIRT1 can directly deacetylate and activate PGC1α [214]. In addition, SIRT1 interacts with multiple transcriptional factors, resulting in enhanced β-oxidation and mitochondrial biogenesis [209]. PPARα is a transcription factor able to bind FAs and increase the expression of genes related to FA catabolism in the mitochondria. In fasting conditions, SIRT1 deacetylates PGC1α, which activates PPAR-α to promote FA oxidation and ATP production [90,215] (Figure 2). Interestingly, SIRT1 transgenic mice have similar phenotypes to mice on a calorie-restricted diet [216]. ATGL positively regulates SIRT1 deacetylase activity to promote PGC1α signaling. ATGL increases LDs lipolysis and the activity of the nuclear receptor PPARα to promote FA oxidation [90]. Liver-specific deletion of SIRT1 resulted in fatty liver, inflammation, and endoplasmic reticulum stress, due to impaired PPARα/PGC1α pathway [215,217]. SIRT1/PGC1α pathway mediates the beneficial effect of antioxidant treatment on mitochondrial function and oxidative stress in hepatocytes [218]. PGC1α increases the expression of ROS detoxifying enzymes such as SOD2, catalase, and antioxidant treatment improves oxidative stress caused by excess fructose via upregulation of SIRT-1 expression [207]. PGC1α, SIRT1, and AMPK represent an energy sensing network that controls metabolic homeostasis [219] (Figure 2). In addition to AMPK, cGMP, endothelial NO synthase, and exogenous NO are all upstream mediators of PGC1α and can increase mitochondrial biogenesis via PGC1α activation [220]. In high energy demands conditions, AMPK is activated by a high AMP/ATP ratio [221]. Once activated, AMPK turns on catabolic pathways to produce ATP while simultaneously turning off energy-consuming anabolic processes. To perform these actions, AMPK quickly regulates metabolic enzymes through direct phosphorylation, but additionally, AMPK can enhance SIRT1 activity by increasing cellular NAD^+^ levels, resulting in the deacetylation of PGC1α. Indeed, AMPK and SIRT1 share common target molecules, including PGC1α, PPARγ, and NF-κB [222,223,224]. Moreover, the treatment of mice fed HFD with resveratrol, a SIRT1 activator, activated the AMPKα-SIRT1 pathway, improved hepatic steatosis, and decreased inflammation [225]. The nicotinamide phosphoribosyltransferase (NAMPT) is a rate-limiting enzyme in NAD^+^ biosynthesis that regulates the activity of NAD^+^-dependent enzymes, such as SIRTs. SIRT1 mediates NAMPT’s effects on lipid metabolism and inflammation [226,227,228,229]. Inhibition of NAMPT aggravated the HFD-induced hepatic steatosis by suppressing the SIRT1-mediated signaling pathway [230]. NAD^+^ precursors improved hepatic mitochondrial function and decreased oxidative stress in Pre-clinical NAFLD models [225]. NAD^+^ repletion, using NAD^+^ precursors such as nicotinamide riboside and nicotinamide mononucleotide, reduced the activation of HSCs and prevented fibrosis and NASH progression. However, initial clinical trials have only shown modest effects when NAD^+^ precursors in obesity [228].

#### 3.2.2. SIRT3 and NAFLD

SIRT3 is a mitochondrial NAD+-dependent deacetylase that regulates the activity of proteins involved in cellular metabolism [201,231]. SIRT3 gene expresses three isoforms, the two long isoforms of murine SIRT3 proteins (M1 and M2) are in the mitochondria. In contrast, the short form of SIRT3 protein (M3) lacks an N-terminal mitochondrial targeting signal in the cytosol. All isoforms have deacetylase activity [232,233,234,235,236]. In fasting conditions, SIRT3 upregulated β-oxidation and ATP production [237], suppressed ROS, and increased mitochondrial biogenesis through activation of PGC1α [238]. Mice deficient in SIRT3 have hyperacetylated mitochondrial proteins [237]. SIRT3 is the highly expressed sirtuin in mouse liver [239]. SIRT3 has been shown to improve mitochondrial function and NAFLD by regulating β-oxidation, ketogenesis, mitophagy, and the antioxidant response system (Figure 3).

SIRT3 is downregulated in human and mouse models of NAFLD ([210] Nassir, 2016 #4485]). SIRT3 and PGC1α can regulate each other, and both are reduced in HFD-fed mice [238]. Downregulation of SIRT3 with HFD feeding in mice induced hyperacetylation of mitochondrial proteins and increased hepatic fat storage and oxidative stress [238,240] (Figure 3). Exposure of mice lacking SIRT3 to HFD further increased the acetylation status of liver proteins and reduced respiratory complexes III and IV activity and increased oxidative stress [241,242]. Palmitate-induced lipotoxicity enhances ROS production and hepatocyte death in SIRT3-deficient primary hepatocytes [243,244]. SIRT3 overexpression reversed the suppression of ATP production induced by palmitate treatment [243,245]. In addition, SIRT3 overexpression repressed ROS generation [241]. HFD feeding in mice lacking SIRT3 exacerbated obesity, insulin resistance, hyper-lipidemia, hepatic steatosis, and inflammation [241,245]. Adenoviral overexpression of SIRT3 in these mice rescued the phenotype [241,242]. In addition to its effect on the mitochondria, SIRT3 deficiency in the liver aggravated hepatic steatosis in HFD-fed mice through upregulation of proteins involved in the FA uptake, such as CD36 and the VLDL receptor [244].

SIRT3 activates multiple targets such as long-chain acyl-CoA dehydrogenase (LCAD) and the acetyl-CoA synthase (AceCS) for acetyl-CoA formation [204]. We have recently that SIRT3 is downregulated in the mitochondrial trifunctional protein heterozygous (MTP^+/−^) mice [239]. Overexpression of SIRT3 in MTP^+/−^ mice deacetylates MTP, increases hepatic levels, and increases mitochondrial function [239]. SIRT3 is a positive regulator of autophagy and macroautophagy [246]. In primary hepatocytes from HFD-fed mice and mouse hepatocytes exposed to palmitic and oleic acid mixture, lipotoxicity decreased SIRT3 expression and lipophagic flux [246]. The decrease in lipophagy further worsened LDs accumulation, eventually leading to severe steatosis and hepatotoxicity. However, SIRT3 overexpression promoted macroautophagy in LDs through activating AMPK [246]. Treatment with Honokiol, a SIRT3 agonist, attenuated hepatic lipotoxicity by promoting SIRT3-AMPK-mediated lipophagy on lipid droplets [246]. In addition to lipophagy, SIRT3 has been shown to regulate mitophagy. Downregulation of SIRT3 with HFD-feeding inhibited Bnip3-mediated mitophagy, causing mitochondria-dependent hepatocyte death [245]. Furthermore, SIRT3 deletion aggravated hepatic steatosis, inflammation, and fibrogenesis in the methionine choline (MCD) mouse model of NAFLD partly by reducing the activity of the antioxidant enzyme SOD2 [247]. SIRT3 over-expression alleviated the MCD-induced phenotype [247], implicating SIRT3 deletion in NASH aggravation with MCD.

SIRTs are a potential therapeutic target for NAFLD as they protect hepatocytes against lipotoxicity [246]. The current state of sirtuin-targeted drug discovery and development has been recently reviewed in [248,249]. Small molecule sirtuin regulators have been developed, with a few compounds targeting human SIRTs still being in clinical development. The fundamental issues are the identification of isoform-specific and site-specific delivery of SIRTs activators [249,250].

## 4. Current NAFLD Diagnosis Methods and Tools

Evidence has suggested that liver fibrosis is reversible at the initial stages. Since fibrosis is the strongest predictor of mortality in patients with MASH, the exact staging of fibrosis and the ability to distinguish NASH from early fibrosis is critical in identifying of patients at risk for developing progressed forms of the disease [251,252]. Both traditional and new tools (imaging and biomarkers) are being used to diagnose and grade the disease, with both advantages and drawbacks.

Blood transaminases are the most performed liver function tests but have not been proven to be dependable and satisfactory in predicting of NAFLD progression [253]. Both abnormal and normal liver enzymes were found in patients with NAFLD [254,255,256]. In addition, decreased alanine aminotransferase (ALT) was found in patients with advanced liver diseases [155,254]. Other biomarkers panels have been used for assessing liver fat, including the Hepatic Steatosis Index (HSI) [257,258], Fatty Liver Index (FLI) [258], and the Steatotest [257], and the Liver Fat Score (LFS) [259]. Non-invasive scoring systems such as Fibrosis-4 (FIB-4), NAFLD Fibrosis score (NFS) [260], Hepamet Fibrosis, Score (HFS), and Platelet Ratio Index (APRI) are used to detect NAFLD progression risk but with modest sensitivity to diagnose early stages of NASH and Fibrosis [253,261,262,263,264]. In addition, a low agreement exists between these fibrosis scoring systems, when applied to the same patient. A comparison between NFS, FIB-4 and HFS in the assessment of the risk of advanced fibrosis showed discordance between the different scoring systems, the strongest agreement was found between FIB-4 and HFS [265].

Liver biopsy and histology remain the gold standard in the diagnosis of NASH. Histologically, NASH is characterized by hepatic steatosis, ballooning, and inflammation with or without fibrosis. Liver biopsy can distinguish between NAFL and NASH [266,267]. However, liver biopsy is an invasive procedure, expensive, with high sampling errors, risk of bleeding, and in rare instances, risk of death [268,269]. In addition, liver biopsy is based on a small liver sample that may not represent the pathology in the rest of the liver tissue. The most common histological scoring systems for NAFLD are the NAS and the steatosis-activity-fibrosis (SAF). NAS evaluates histologic steatosis, lobular inflammation, hepatocellular ballooning, and fibrosis [270]. The NAS is the unweighted sum of semiquantitative scores for steatosis (0–3), lobular inflammation (0–3), and hepatocellular ballooning (0–2) [270]. The SAF is defined as the addition of the ballooning and lobular inflammation, weighted equally and scored from 0–2 [271]. NAS and SAF show high agreement in diagnosing definite NASH. However, about 50% of patients characterized as ‘borderline‘ by NAS had a ‘definite NASH according to SAF score [272]. Since early detection of NASH may help prevent fibrosis, there is a need for minimally invasive imaging tools and biomarkers. The focus in the field is on finding non-invasive, reproducible biomarkers for the evaluation of NAFLD, its progression risk, and the validation of treatments in clinical trials.

Plasma cytokeratin 18 (CK18) fragment levels are one of the most used biomarkers for hepatocyte injury. However, the CK18 biomarker suffers from moderate accuracy and variability [273,274]. The sensitivity and specificity of the CK18 biomarker could be improved when combined with other biomarkers such as adiponectin, resistin, and IL 6 [273,274]. Inflammation-related circulating markers, including cytokines chemokine or shed receptors from immune cells, and circulating exosomes related to inflammation have been proposed as scoring tools for NAFLD but lack validation studies [275,276].

Ultrasound (US) is the first-line imaging test used in clinical practice, in individuals with suspected NAFLD, with a typical appearance of a hyperechogenic liver. Ultrasound is being used to detect liver fat but only detected moderate-to-severe steatosis (more than 20%) [277]. In addition, the sensitivity of US for hepatic steatosis evaluation is affected by the presence of severe fibrosis. The computed-assisted US hepatic/renal ratio (H/R) and US hepatic attenuation rate are used to assess early steatosis. Attenuation imaging is a novel approach for detecting hepatic steatosis through ultrasound imaging, providing convenience for routine screening of liver fat [278].

Magnetic resonance spectroscopy (MRS) is the most accurate non-invasive method to quantify liver fat. MRS measures proton signals as a function of their resonance frequency to separate fat and water signal fractions. Magnetic resonance imaging proton density derived fat fraction (MRI-PDFF) is the leading tool for evaluating fat content in the liver and has been validated against liver histology [279,280]. A recent study indicated that a 30% relative reduction in liver fat content assessed by MRI-PDFF might be associated with histologic improvement in NASH. Nevertheless, the procedure is not patient-friendly, expensive, and the instrument is not widely available [281].

Transient elastography (TE) is an ultrasound-based system that evaluates liver fibrosis using Fibroscan with an M probe. Controlled attenuation parameter (CAP) is a non-invasive assessment of steatosis, concomitantly to fibrosis, using Fibroscan [277]. In CAP, liver fat is quantified by measuring the attenuation of a US beam in the liver. An XL probe was designed to overcome the lower accuracy of the M probe in differentiating hepatic steatosis and fibrosis in obese people. However, even though the XL probe improved the rate for assessing hepatic steatosis and fibrosis in obese patients, the rates are still low in obese people with BMI ≥ 30 [282]. A strong correlation has been found between liver stiffness and liver fibrosis or scarring. The utility of the Fibroscan in predicting significant liver fibrosis is limited in morbidly obese subjects [283]. Fibrotouch liver elastography is a new tool to assess liver fibrosis that appears to be cost-effective, simple, and evaluates fibrosis in all patients irrespective of obesity [284].

Magnetic resonance elastography (MRE) estimates liver stiffness [277]. MRE is the most accurate non-invasive MRI-based technique to evaluate liver stiffness and stage of fibrosis, independent of BMI. The use of MRE is limited by cost, availability, and time for exams [264,277].

## 5. Emerging Biomarkers

The need to develop non-invasive biomarkers to differentiate simple steatosis from NASH and NASH to early fibrosis is increasing. This section will discuss the role of non-coding RNA as an emerging non-invasive biomarker for NAFLD.

Most human RNA transcripts do not encode for proteins. Non-coding RNAs (ncRNAs) include short RNAs (<30 nucleotides) such as microRNAs (miRNA) and long noncoding RNAs (>200 nucleotides) such as circular RNAs (circRNAs) [285,286,287]. nRNAs regulate cell physiology and functions through epigenetic gene silencing by post-transcriptionally regulating mRNA stability. The abnormal expression of ncRNAs has been associated with pathologies such as NAFLD [288,289,290]. In addition, the role of exosome miRNAs in NAFLD has recently attracted attention [289].

Circulating miRNAs, including miR-122, miR34, miR-192, and miR-375, were upregulated in NAFLD and were positively correlated with the disease severity [291,292]. NASH patients showed a systematic downregulation in these miRNAs in the liver and upregulation in serum [292,293]. The most notable change was seen for miR-122, the most abundant and liver-specific miRNA (more than 70% of the total liver miRNA pool) [291]. miR-122 was significantly upregulated in NAFLD patients’ serum and was suggested as a potential biomarker for NAFLD and its progression [293,294,295,296]. Knockdown of miR-122 inhibited lipid production and suppressed the expression of lipogenic genes, in free FA-treated human hepatoma cells, via upregulating SIRT1 [297]. miR-122 expression correlated with NAFLD incidence in obese children [298]. Notably, the hepatic miR-122 level is positively correlated with histopathological features in NAFLD patients [293,295,298,299]. In vitro, inhibition of miR-122 in liver organoids resulted in steatosis, inflammation, necrosis, and fibrosis [299]. The inhibition of miR-122 protects hepatocytes from lipid metabolic disorders such as NAFLD and suppresses lipogenesis via elevating SIRT1 and activating the AMPK pathway [297]. Downregulation of miR-34c modulates NAFLD by regulating SIRT1 and PPARα [300]. miR-378 plays a key role in hepatic inflammation and fibrosis by positively regulating the NF-κB-TNFα axis. miR-378 reduced SIRT1 activity and facilitated an inflammatory pathway involving NF-κB-TNFα [301]. The expression of miR-421 was significantly upregulated in the liver in a mouse model of NAFLD to modulate lipid metabolism and oxidative stress. Overexpression of miR-421 decreased SIRT3 and FOXO3 protein levels and increased oxidative damage by reducing SOD and catalase activity [302].

Interestingly, mitochondrial circRNAs account for a substantial fraction of downregulated circRNAs in fibroblasts from patients with NASH. One example is the steatohepatitis-associated circRNA ATP5B Regulator (SCAR). By constructing mitochondria-targeting nanoparticles to assure delivery to the mitochondria, Zhao et al. found that SCAR inhibits mitochondrial ROS production and fibroblast activation [303].

lncRNAs are abnormally expressed in oxidative stress-related liver diseases, including liver fibrosis [304] and HCC [305]. lncRNAs have been recently proposed to play a role in the pathogenesis of NAFLD and its progression [306,307,308,309]. The mitochondrial permeability transition pore (mPTP) has a significant role in mitochondrial homeostasis. Excessive opening of mPTP leads to mitochondrial stress, including impaired mitochondrial ETC function, mitochondrial swelling, and ROS generation [310]. Cyclophilin D (CypD), an intramitochondrial peptidylprolyl-cis-trans-isomerase, is an initial factor of the mPTP [311]. Increased expression or enhanced activation of CypD leads to excessive opening of the mPTP [312]. Acetylation of CypD stimulates excessive mPTP opening and oxidative stress [312]. Oxidative stress-related liver diseases were associated with elevated levels of serum thyroid-stimulating hormone (TSH). Wang et al. [313] found that TSH knockout mice had much higher lncRNA-AK044604 expression than their littermate counterparts. TSH downregulated the expression of the lncRNA-AK044604 and SIRT1/SIRT3 deacetylase activity, leading to CypD acetylation and increased mitochondrial stress [313]. The role of lncRNAs in liver disease is still not fully understood. One of the suggested functions of lncRNA is its role as a miRNA sponge and can prevent miRNA actions toward the target mRNAs. This co-regulatory network between lncRNA and miRNA may add to the complexity of the relationship of miRNAs to their targets [309].

## 6. Conclusions and Perspective

Progress has been made in understanding the pathophysiology of hepatic steatosis and NASH. Nevertheless, NASH is still a growing disease without pharmacological treatment. The recent important question is how NASH develops and progresses to fibrosis, the strongest predictor of mortality in NAFLD patients. Lifestyle interventions have a positive impact on NAFLD. However, the underlying mechanisms and the implementation of lifestyle changes as a medical strategy are unclear and might not apply to every NAFLD patient. Liver biopsy is still the gold standard for assessing liver health with sampling limitations and errors. Identification of accessible non-imaging tools and accurate biomarkers will help, in clinical trials, to validate emerging treatments. There is still an unmet need for reliable biomarkers and non-invasive and inexpensive tools to accurately stage the progression of NAFLD and validate the safety and efficacy of potential treatments.
